# Retraction: Suppression of AP1 Transcription Factor Function in Keratinocyte Suppresses Differentiation

**DOI:** 10.1371/journal.pone.0247222

**Published:** 2021-02-11

**Authors:** The PLOS ONE Editors

The University of Maryland, Baltimore, has investigated the work reported in this article [1]. The investigation committee recommended retraction of the article and concluded that it is compromised in light of their findings about Figs 1 and 2. Specifically:

- In Fig 1B, the TAM67-Flag panel shows an image that is labelled in the raw data records as results of a Cyclin western blot experiment. Furthermore, a band visible on the original blot appears to have been deleted from the published image.
- Fig 1C shows a composite image in which two micrograph images were spliced together.
- In Fig 2C, the panels representing junB and junD do not include the indicated experimental data. Instead, they show empty areas of blot films away from the area that captured proteins of the expected molecular size for junB and junD.
- In Fig 2C, the TAM67-FLAG panel shows results of a Fra-1 western blot experiment, and a band visible on the original blot appears to have been deleted from the published image.

In light of the above concerns and in line with the University’s recommendation, the *PLOS ONE* Editors retract this article.

EAR, WX, and RLE did not agree with retraction. The other authors either could not be reached or did not respond directly.
